# Spermatozoa centriole quality determined by FRAC may correlate with zygote nucleoli polarization—a pilot study

**DOI:** 10.1007/s10815-025-03411-x

**Published:** 2025-02-07

**Authors:** Derek F. Kluczynski, Ankit Jaiswal, Min Xu, Nagalakshmi Nadiminty, Barbara Saltzman, Samantha Schon, Tomer Avidor-Reiss

**Affiliations:** 1 Department of Biological Sciences, College of Natural Sciences and Mathematics, University of Toledo, Toledo, OH USA; 2 Division of Reproductive Endocrinology and Infertility, Department of Obstetrics and Gynecology, University of Michigan, Ann Arbor, MI USA; 3 Department of Urology, College of Medicine and Life Sciences, University of Toledo, Toledo, OH USA; 4 Department of Population Health, College of Health and Human Services, University of Toledo, Toledo, OH USA

**Keywords:** Spermatozoa, Centriole, Nucleolus Precursor Body, Reproduction, Zygote, Infertility

## Abstract

**Purpose:**

Spermatozoa centriolar defects can result in abnormal zygote functions. Recently, a method to quantify spermatozoa centriolar defects was developed named Fluorescence-Based Ratiometric Analysis of Sperm Centrioles (FRAC). However, whether spermatozoa centriolar defects identified by FRAC can result in abnormal zygote functions was not tested.

**Methods:**

Here, we quantified spermatozoa centriolar defects using FRAC, and zygote centriole function was assessed by imaging Nucleolus Precursor Body (NPB) polarization which was based on the pattern of NPB polarization. Data was analyzed at couple and embryo levels. Subjects were divided into two groups: seven couples and 62 embryos with normal spermatozoa centrioles versus eight couples and 78 embryos with abnormal spermatozoa centrioles (140 embryos from 15 couples in total).

**Results:**

Patterned NPB polarization was statistically significant in both couple- and embryo-level analyses (*p* < 0.0001 and *p* = 0.0024). These results suggest that the abnormal spermatozoa centrioles identified by FRAC may correlate with abnormal zygote centrosome function via NPB polarization scoring.

**Conclusions:**

This study provides a foundation for more extensive studies to test for FRAC’s utility in assessing spermatozoa centriole quality.

**Supplementary Information:**

The online version contains supplementary material available at 10.1007/s10815-025-03411-x.

## Introduction

Infertility is the inability to conceive a child after 6 or 12 months of unprotected sexual intercourse, depending on the female partner’s age [1], impacting one in seven couples [2]. Couple infertility can be broken into three factors: male infertility factors make up approximately a third of the causes of infertility, female infertility factors make up another third, leaving the last third to be unexplained infertility factors [3, 4]. These unexplained factors may derive from either partner or a combination. Here, we explore a critical but understudied spermatozoa factor with an emerging role in currently unexplained infertility: the centriole.

Tests used clinically to determine male infertility are divided into basic, extended, and advanced examinations based on the tests’ use rates and credentials [5]. Basic tests commonly determine infertility factors, such as abnormal spermatozoa concentration, morphology, and motility [6]. Extended tests examine factors such as spermatozoa DNA fragmentation and aneuploidy, while advanced examinations include testing for factors such as oxidative stress and acrosome reaction [7]. Another category of frontier tests under consideration is the experimental examinations, which include the examination of the critical spermatozoa organelle, the centriole [8–12].

**Fig. 1:**
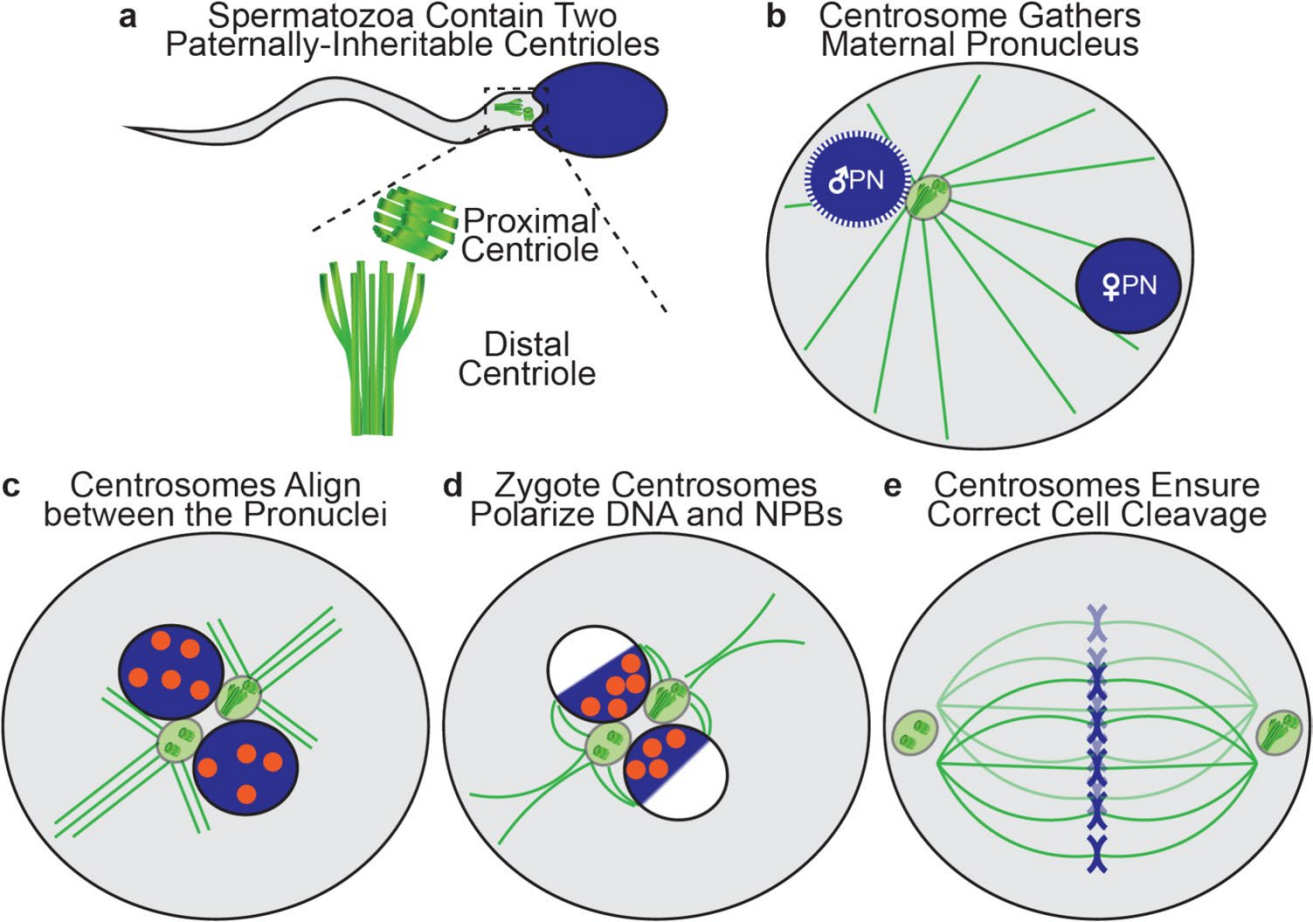
Schematic of zygote centriole dynamics: **a** The spermatozoon neck contains two centrioles: the canonical proximal and the atypical distal centrioles. **b** The fertilized egg inherits a paternal pronucleus (blue with dashed line) and two centrioles (green dots). The centrioles form a centrosome and send out a microtubule aster (green lines) to pull the maternal pronucleus (blue) towards the paternal pronucleus. **c** The two pronuclei now sit in between the inherited centrosome and the newly formed centrosome with Nucleolus Precursor Bodies (orange) appearing in the two pronuclei. **d** The two centrosomes interact with the pronuclei’s nuclear pores and polarize the DNA and Nucleolus Precursor Bodies to prepare for the first cleavage. **e** The two centrosomes associate with the dual spindles poles, helping to organize and ensure correct cell cleavage [32]

Human spermatozoa and other non-murine mammals have two distinct centrioles in the neck (aka connecting piece or head–tail coupling apparatus), which connect the tail to the head [10, 13, 14] (Fig. 1a). The proximal centriole is located closer to the head and is barrel-shaped, similar to the canonical somatic cell centriole. The distal centriole is closer to the tail and has an atypical structure and composition [15]. It develops into a fan-shaped structure during spermiogenesis and is part of a dynamic complex connecting the tail to the head [16]. The distal centriole also forms the flagellum of the spermatozoa [17]. Centriole abnormalities, such as a mutation in CEP135 [18] and POC1B [19], usually affect the spermatozoon flagellum morphology. However, there are some cases where an abnormal centrosomal protein does not indicate an apparent flagellum abnormality, such as CEP112, but causes acephaly, the separation of the spermatozoon head from the tail [20]. The human spermatozoa centriole structure represents the mammalian ancestral form, distinct from the remnant centriole in mice [21–23].

To determine the contribution of spermatozoa centrioles to male infertility factors, we developed a novel assay to measure spermatozoa centriole quality through quantitative protein analysis called Fluorescence-Based Ratiometric Analysis of Sperm Centrioles (FRAC) [24]. We have found that this assay can measure spermatozoa centriole protein relative amounts in humans and bovines [25, 26]. FRAC measures selected proteins in three locations: the proximal centriole, the distal centriole, and the axoneme, producing, based on these locations, three parameters for each protein measured. Spermatozoa and their centrioles are compared to a reference population of males with proven fertility. If all parameters measured for a male are inside the reference range, they are considered to have a normal FRAC value of zero (the test is negative). If there is any parameter that is outside of the reference range, that infertile male is considered to have an abnormal FRAC value of one or more and, therefore, classified as having abnormal spermatozoa centriole quality (the test is positive). We have shown that FRAC can identify some males in couples with unexplained infertility as having male-factor infertility [26] and unexplained male subfertility in bovine [25]. While FRAC can measure spermatozoa centriole quality, it is unknown if males with abnormal centrioles, as defined by positive FRAC values, also demonstrate subsequent zygote abnormalities.

The zygote inherits centrioles only from the spermatozoa [27–29] (Fig. 1a). Together with centriole-recruited pericentriolar protein from the egg, these centrioles form the zygote’s first centrosomes [30]. The two centrioles are initially near each other, creating one centrosome associated with a decondensed spermatozoon pronucleus (Fig. 1b). This centrosome assists in bringing the female and male pronuclei together. Later, after the male and female pronuclei are adjacent (i.e., in opposition), the male pronucleus rotates, placing the two centrioles—now separated into two centrosomes (four centrioles in total)—at the pronuclei junction [31] (Fig. 1c). At this time, the centrosomes polarize the pronuclei content, setting critical interactions with the chromosomes (Fig. 1d). Later, as the male and female spindles are formed and aligned, the centrosomes associate with the spindle’s poles, ensuring the embryo has the correct cell division axis [32, 33] (Fig. 1e). Abnormal centrosome function leads to anomalous cleavage with an abnormal number of chromosomes in the blastomeres [11, 34]. This anomaly can ultimately lead to the death of the zygote, morula, or blastula [12, 35, 36], suggesting that spermatozoa centriole dysfunction results in infertility.

In addition to forming the spermatozoon asters and polarizing the pronuclei’s chromosomes, the zygotic centrosomes interact via the asters and the pronuclei pores with the pronuclei Nucleolus Precursor Bodies (NPBs) [37] (Fig. 1c). NPBs are prominent and morphologically distinct nucleoli that are transcriptionally inactive and appear during the paternal and maternal pronuclei formation about six hours post-fertilization [38, 39]. NPBs are associated with chromatin and can be used as a proxy for chromosome location [40]. Most importantly, NPBs can be identified by simple light microscopy, and NPB polarization can inform researchers and clinicians about zygotic centrosome quality, which is expected to correlate with spermatozoa centriole quality and pericentriolar material that is recruited by the centrioles after fertilization. This study tested whether centriolar defects determined by a positive FRAC score correlate with abnormal zygotic NPB polarization.

## Materials and methods

We analyzed spermatozoa samples and embryo videos using FRAC and NPB polarization. The FRAC assay utilized in this study was previously described [24–26], while the NPB polarization method was adapted from previous techniques [36, 37, 41].

As there is no standard methodology to assess NPB polarization, we quantitatively evaluated NPB polarization by studying the pattern of NPB polarization. We analyzed patterned NPB polarization at two levels: (i) based on a couple population (*n* = 15) and (ii) based on an embryo population (*n* = 140). The couple population refers to the combined male and female partners seeking infertility treatment. Patterned NPB polarization had one hypothesis at the couple- and embryo-level analyses.

Through our studies, we explored four statistical hypotheses related to FRAC correlation with NPB polarization (Online Resource 1). Therefore, we corrected our critical *p*-value (*α*) using the Bonferroni correction method to 0.0125 (0.05/4) in our hypothesis. All figures were created using Adobe Photoshop and Adobe Illustrator.

### Differential gradient centrifugation, washing, attachment, and fixation

Semen samples were obtained from the Reproductive Subject Registry and Sample Repository (RSRSR) at the University of Michigan and were thawed before use. The semen samples were separated into interface and pellet spermatozoa fractions using differential gradient centrifugation and PureSperm®. In this study, only pellet spermatozoa fractions were used and analyzed. In a conical tube, 1 mL of PureSperm® 80% (Nidacon, PS80-100) was placed at the bottom, 1 mL of PureSperm ®40% (Nidacon, PS40-100) was pipetted onto the PureSperm® 80%, and finally, the semen sample from a patient was pipetted on top of the PureSperm® 40%. The conical tube was centrifuged for 20 min at 400 × g, and the supernatant was discarded with a pipette, leaving only the pellet. The pellet was then washed with 2 mL of PureSperm® Wash media (Nidacon, PSW-100) and resuspended using a pipette. The conical tube was then centrifuged for 8 min at 250 × g, with the supernatant being removed, leaving only the pellet. This pellet was resuspended using a pipette with 100 μL of Medium 199 media (Sigma-Aldrich, M7528). All PureSperm® media and Medium 199 media were brought to 37 °C before use.

Samples were then evenly aliquoted between 15 glass slides (Azer Scientific, EMS200A +), approximately 10 μL per aliquot. Glass coverslips (VWR, 48366–205) were placed over each aliquot. Slides were then snap-frozen and stored in liquid nitrogen until used for spermatozoa labeling.

Spermatozoa samples were prepared and analyzed as described by Jaiswal and colleagues [26].

### Spermatozoa labeling

The spermatozoa population from each infertile male was analyzed and labeled three independent times. Coverslips were removed with forceps, and the sample slides were fixed in ice-cold (− 20 °C) methanol for 5 min. The slides were then washed in 1X PBS for 1 min, followed by a permeabilization solution of 1X PBS with 0.3% Triton X-100 (Sigma-Aldrich, 9002–93-1) for 1 h. They were then blocked with 1% BSA (CHEM-IMPEX INT’L, 00535) in 1X PBS with 0.3% Triton X-100 for 30 min.

A solution of 200 μL of primary antibodies (Online Resource 2) diluted in 1X PBS with 1% BSA and 0.3% Triton X-100 was applied to each slide, covered with Parafilm (Bemis, 13–374-12), and incubated overnight at 4 °C. After incubating, each slide was washed in 1X PBS with 0.3% Triton X-100, three times for 5 min each. After this wash, a solution of 200 μL of secondary antibodies (Online Resource 2) diluted in 1X PBS with 1% BSA and 0.3% Triton X-100 was applied to each slide, covered with Parafilm, and incubated at room temperature for 1 h. The slides were washed in 1X PBS with 0.3% Triton X-100 three times for 5 min each. Finally, the slides were washed in 1X PBS three times for 5 min each and prepared for mounting. One drop of Fluoroshield with DAPI (Sigma-Aldrich, F6057) was added to the slide, where a glass coverslip (Thermo Scientific, 16940) was placed over the sample. It was sealed with clear nail polish (EMS Diasum, 72180) and stored at − 20 °C until imaging. Spermatozoa samples were labeled as described by Jaiswal and colleagues [26].

### Confocal microscopy

Image acquisition of spermatozoa samples for FRAC analysis used a Leica SP8 confocal microscope in photon counting mode. The fluorescence signal was collected in four sequences. Sequence one collected two images: DNA and phase-like. Sequence two collected one image: acetylated tubulin. Sequence three collected one image: tubulin. Sequence four collected one image: POC1B. Images were captured with 10 to 20 Z-sections of 0.3-μm thickness per spermatozoon. Confocal microscopy was performed as described by Jaiswal and colleagues [26].

### Labeling quantification

Quantification for FRAC was performed by two raters blinded to the identity of the quantified spermatozoa. Raters used the Leica Application Suite X (LasX) program. For each image, a max projection of multiple Z planes was used, and each spermatozoon was identified with regions of interest (ROIs) (0.5 × 0.75 μm rectangles) being placed in the three locations studied: the proximal centriole, the distal centriole, and the axoneme. Only viable spermatozoa were quantified and had ROIs drawn. Viable spermatozoa included those with a clear and unobstructed view of the neck and tail. The first ROI was drawn on where the proximal centriole is, adjusting the box angles to contain the highest amount of fluorescence. The second ROI was drawn on where the distal centriole should be, according to the highest amount of fluorescent intensity. The third ROI was drawn two μm below the bottom of the border of the distal centriole ROI along the axoneme. This third ROI was drawn as a control, and the two μm length is arbitrary. The human distal centriole is about 230 nm in width by 186 nm in length [15]. Labeling quantification was done as described by Jaiswal and colleagues [26].

### FRAC analysis

While quantification values derived from the Leica Application Suite X program can vary due to many factors, FRAC analysis addresses this by normalizing the variability for each spermatozoon analyzed. This is done by creating a ratio for each of the analyzed locations. We divide the protein fluorescence of an individual ROI by the total of the three ROIs. This generates the ratio that we call the FRAC ratio.

Statistical analysis is done in Microsoft Excel, where LasX pixel sum data for each ROI is copied. For example, for each spermatozoon, we divide the ROI that labels the proximal centriole by the total ROI pixel sum data that labels the proximal centriole, distal centriole, and axoneme. We then do this for the ROI pixel sum data for both the distal centriole and axoneme for each spermatozoon. We then aggregate each FRAC ratio for each spermatozoon and combine them to create a mean FRAC ratio for that male patient. Fertile male patients and their spermatozoa make up the reference population, and infertile male patients and their spermatozoa make up the experimental study population. The reference range comprises the mean FRAC ratio ± two standard deviations. An experimental male patient range comprises their mean FRAC ratio and a 95% confidence interval. If they fall within the reference range, they are considered normal FRAC values, and those outside the reference range are considered abnormal FRAC values.

### Nucleolus precursor body polarization scoring

Embryo videos were captured by the University of Michigan researchers using the EmbryoScope Plus time-lapse system. The time-lapse pictures were taken every 10 min for each embryo in 11 focal planes. The middle five focal planes, where the pronuclei are present, are used for studying Nucleolus Precursor Body (NPB) polarization. We determined the clearest focal plane from these videos to quantify the NPBs for each video. As NPBs can be of varying sizes and since there are different focal depths, we counted any large or small discrete circular body in the pronuclei as an NPB. We analyzed every patient embryo, ranging from 3 to 17 embryos per patient (9.0 ± 3.7). EmbryoScope Plus videos were taken between November 2018 and April 2021.

To study the pattern of NPB polarization (patterned Nucleolus Precursor Body polarization scoring), we analyzed NPB polarization scores (see equation in the “Results” section) throughout the entire zygote development. Given embryo video constraints, this method looked as early as possible (~ 15 h of zygote development) to as late as the last recorded NPB disappearance just before the first cleavage (~ 40 h of zygote development). This included taking an NPB polarization score from the appearance to the disappearance of the NPBs. Since this considered multiple time points and not just one, there are times when an NPB polarization score could not be calculated due to a significant overlap between the two pronuclei, unfocused images where NPBs could not be counted, or other issues that obscured NPB counting.

### Insemination, embryo culture, and time-lapse annotation

Oocytes were inseminated either through conventional in vitro fertilization (IVF) or intracytoplasmic sperm injection 4 to 5 h after retrieval. A fertilization check was carried out within 17 h after insemination, and a zygote with two pronuclei was considered normally fertilized. The normally fertilized embryos were cultured uninterruptedly in EmbryoScope Plus up to 6 days post-retrieval at 37 °C, 5.5% CO_2_, and 5% O_2_. According to the Gardner system, the embryos were evaluated for inner cell mass and trophectoderm on day 5 or 6 [42]. The blastocysts at or above stage three with an apparent inner cell mass were scored as healthy blastocysts. The blastocysts at stages one and two were not considered blastocysts in the setting of this study.

### Demographic information

The average male was 33.0 ± 3.7 years old, and the average female was 32.2 ± 3.7 years old. In normal FRAC couples, the average male age was 33.8 ± 4.8 years, and the female age was 33.1 ± 4.0 years. In abnormal FRAC couples, the average male age was 32.2 ± 2.3 years, and the female age was 31.3 ± 3.5 years. Eight of the 15 males had a male-factor infertility diagnosis. The 15 patients with normal and abnormal FRAC had similar average semen parameters. No statistically significant differences were found in age, spermatozoa number, spermatozoa volume, spermatozoa concentration, spermatozoa motility, spermatozoa progression, or spermatozoa morphology (Table 1). Thirteen of the 15 females had some female-specific infertility diagnosis, including ovulatory dysfunction, diminished ovarian reserve, polycystic ovary syndrome, endometriosis, recurrent pregnancy loss, and tubal disease. Individual infertility diagnoses and male/female age can be found in Online Resource 3.

**Table 1:** Semen parameters of patients with normal and abnormal FRAC were similar

	Normal FRAC Range, mean ± SD, total *N*	Abnormal FRAC Range, mean ± SD, total *N*	*p*-value by *T*-test
Age (years)	26.7–39.1, 33.8 ± 4.8, 15	29.3–36.1, 32.2 ± 2.4, 15	0.422
Spermatozoa number analyzed (FRAC)	71–127, 107 ± 18, 752	79–127, 103 ± 13, 826	0.613
Spermatozoa volume (mL)	2.5–8.3, 4.6 ± 2.2, 32	2.5–6, 3.6 ± 1.2, 29	0.310
Spermatozoa concentration (× 10^6)	8–165, 74.3 ± 48.5, 15	2–308, 93.3 ± 103.2, 15	0.665
Motility (%)	36–67, 50.4 ± 11.2	29–56, 42.9 ± 10.2	0.193
Progression (%)	0–62, 40.1 ± 20.1	20–51, 32.0 ± 12.7	0.360
Morphology (%)	2–14, 6.7 ± 4.4	2–18, 5.6 ± 5.3	0.673

### Statistical analysis

Microsoft Excel was used to analyze the FRAC and NPB polarization data statistically. The Microsoft Excel T-TEST function was used to calculate the *p*-values where indicated. A chi-square test of independence ((*O*-*E*)^2/*E*) was used to calculate the *p*-values of the patterned NPB polarization at the couple- and embryo-level populations using the function CHISQ.DIST.RT. This test was tested between the qualitative FRAC and the quantitative NPB polarization rates. All numbers were rounded to the thousandth place, and normal rounding was used. IBM SPSS Statistics (Version 29.0.2.0) was used to calculate the receiver operating characteristic (ROC) curve.

## Results

### FRAC assay identifies a subgroup of infertile males with reduced centriole quality

We selected 15 males with different forms of infertility who underwent infertility treatments with either IVF or ICSI. Six male patients had a normal semen analysis, and nine had male factor infertility (Online Resource 3). Specifically, for semen analysis, two patients had asthenozoospermia, three patients had asthenoteratozoospermia, one patient had teratozoospermia, one patient had oligoteratozoospermia, one patient had oligoasthenozoospermia, and one patient had oligoasthenoteratozoospermia. Semen samples were analyzed through Fluorescence-Based Ratiometric Analysis of Sperm Centrioles (FRAC) using the three previously described biomarkers: POC1B, acetylated tubulin, and tubulin [26]. In total, 1578 spermatozoa were quantified, ranging from 71 to 127 per patient (105 ± 15). The FRAC findings from the 15 infertile patients were compared to the standard obtained from fertile males collected by the RSRSR biorepository described in our previous study by Jaiswal and colleagues [26].

**Fig. 2:**
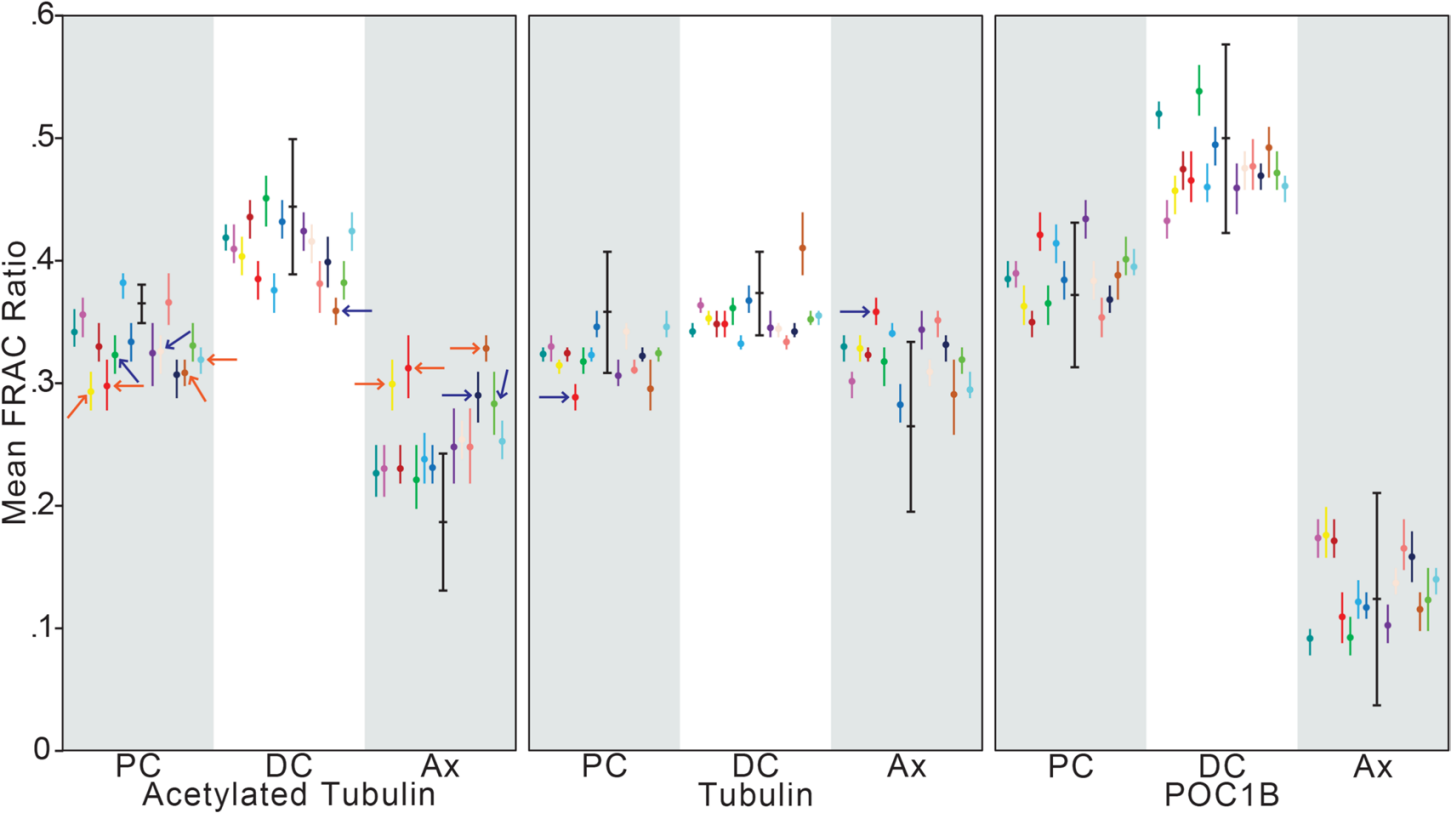
The studied population had seven couples with negative FRAC values and eight with positive FRAC values. Graph depicting the average FRAC ratio from each male (± 95% confidence intervals) for the nine parameters (for example, acetylated tubulin PC is a parameter). Each parameter shows male patients 1 to 15 from left to right. Black bold bars at the center of each group of confidence intervals represent the reference average plus/minus two standard deviations. Arrows point at 95% confidence intervals up to one standard deviation (blue arrows) or more than two standard deviations (orange arrows) outside the reference range

The comparison identified seven infertile males with no outlier values and, therefore, with a negative (i.e., normal) FRAC score. We found eight infertile males with at least one outlier each and, thus, with positive (i.e., abnormal) FRAC values. Of the three proteins analyzed, acetylated tubulin had 12 outliers, with six in the proximal centriole, one in the distal centriole, and five in the axoneme. Tubulin had only two outliers, one in the proximal centriole and one in the axoneme. POC1B, a centriole-specific protein, had no outliers (Fig. 2). Similar profiles of outliers, where acetylated tubulin is the most common, were identified in our previous studies in humans and bovine [25, 26].

### Nucleolus Precursor Body polarization scoring was determined based on five zones

During zygote progression towards cleavage, pronuclei NPB transition from evenly distributed to enrichment at the pronuclei edges facing each other [36, 41]. We quantified NPB polarization in videos generated in the clinical setting from ICSI/IVF embryos based and adapted on the approach described by Cavazza and colleagues [37], Scott [36], and Tesarik and Greco [41]. We excluded from this calculation any embryo where a conclusive determination of polarized NPBs was impossible or if the video started too late to capture the two pronuclei (2PN) stage.

**Fig. 3:**
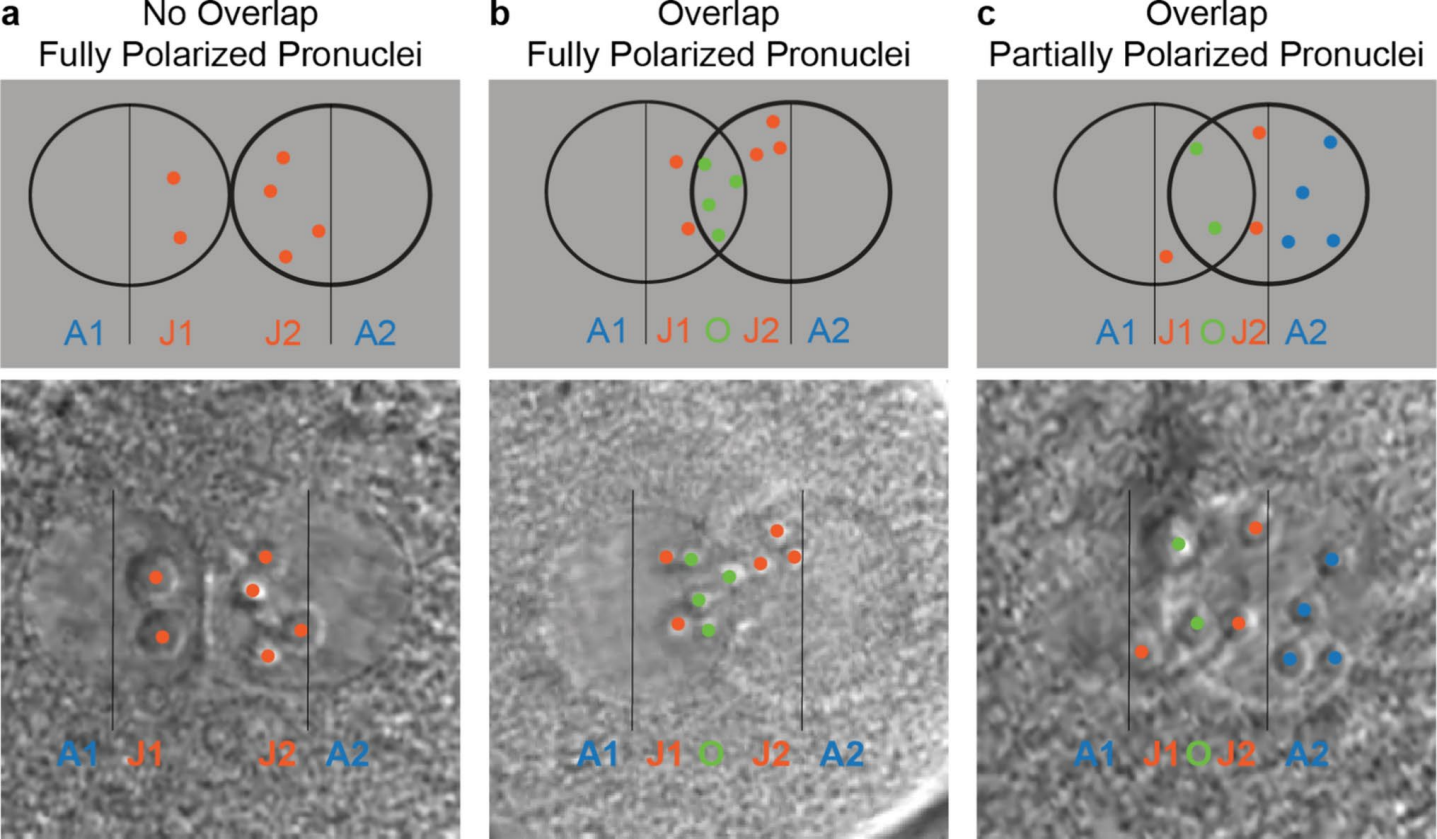
Three scenarios of NPB polarization in the pronuclei of zygotes. **a–c** Represented model (top row) and annotated image (bottom row). Each pronucleus was divided into two halves, separated into either an “A” zone, the half that was furthest away from the junction, or a “J” zone, the half that was towards the junction; **a** a scenario of full NPB polarization in the two non-overlapping pronuclei. There were six NPBs (orange) in total, all in either J1 or J2 zones; **b** a scenario of full NPB polarization in the two overlapping pronuclei. There were nine NPBs in total, with five being in either J1 or J2 zones and four NPBs (green) being in the “O” zone, the zone created by the overlap of the two pronuclei; **c** a scenario showing partial NPB polarization in the two pronuclei with overlap. There were nine NPBs in total, with three NPBS being in the “J1” or “J2” zones, two NPBs in the “O” zone, and four NPBs (blue) being in the “A2” zone

To quantify Nucleolus Precursor Body polarization, we divided each pronucleus into two zones: the half towards the junction (J) and the half away from the junction (A) (Fig. 3). Although the ideal conditions for observing pronuclei were when they were side-by-side with no overlap, that was often not the case in clinical videos. The two pronuclei overlap in most cases, creating an Overlap (O) zone. These divisions made five scorable zones: A1, J1, O, J2, and A2. Zones J1 and J2 were the pronuclei halves where the two pronuclei meet at their junction, and NPBs in these zones counted as polarized. Zone O was the two pronuclei overlap location, with NPBs counting as polarized here as well. Zones A1 and A2 were the pronuclei halves away from the junction, and NPBs in these zones counted as nonpolarized. If the overlap was more than 50% for one or both pronuclei of an embryo, their NPBs were not counted, and that embryo was excluded from analysis. We considered the center of the NPB if it was at the border of A and J zones or the border of J and O zones. For each embryo, we assigned an NPB polarization score using the equation below:

$$\text{Percent of Polarized NPBs}=\frac{\text{J}1+\text{O}+\text{J}2}{\text{A}1+\text{J}1+\text{O}+\text{J}2+\text{A}2}*100$$

A polarization score at or above 91% was counted as polarized, and anything below 91% was classified as non-polarized. This 91% cutoff was obtained from pre-analysis, where over half of abnormal FRAC couples were above the cutoff (Online Resource 4). NPBs were most polarized anywhere from 18.5 to 25.6 h (21.9 ± 2.1 h) after fertilization and 1.8 to 8.9 h (4.5 ± 2.0 h) before pronuclear breakdown. Additionally, the average amount of NPBs in an embryo belonging to a normal FRAC couple was 9.1 ± 2.5, and to an abnormal FRAC couple was 11.4 ± 3.5 (*p* < 0.0001, *T*-test).

### Three patterns of NPB polarization progression were observed in the zygote

The patterned NPB polarization approach calculated the NPB polarization rates from the first NPB appearance to their disappearance shortly before the first cell division in 10-min frame intervals. We visualized zygote centrosome function from as early as 15 h post-fertilization (18.1 ± 2.0) up to 40 h (25.6 ± 3.9) post-fertilization. This comprehensive approach allowed for a pattern of NPB polarization formation to be seen (Online Resource 5). Three different patterns were observed:

1. Full polarization patterns with consistently high NPB polarization rates above or equal to 91% were observed in 44 embryos.
2. Eventual polarization patterns in which NPB polarization rates rose from low (below 91%) to high (equal to or greater than 91%) were observed in 35 embryos.
3. Reduced polarization patterns with consistently low NPB polarization rates (below 91%) were observed in 61 embryos.

An interesting pattern was observed in one embryo from couple 6, where it went from highly polarized (≥ 91%) to low-to-mid-polarized (< 91%). Additionally, in the reduced polarization patterns, 11 embryos had pronuclei go above the 91% polarization cutoff during polarization but still started and ended well below the cutoff. Eight of these embryos belonged to abnormal FRAC couples, and only three belonged to normal FRAC couples.

### Some couples with abnormal FRAC have reduced NPB polarization

**Fig. 4:**
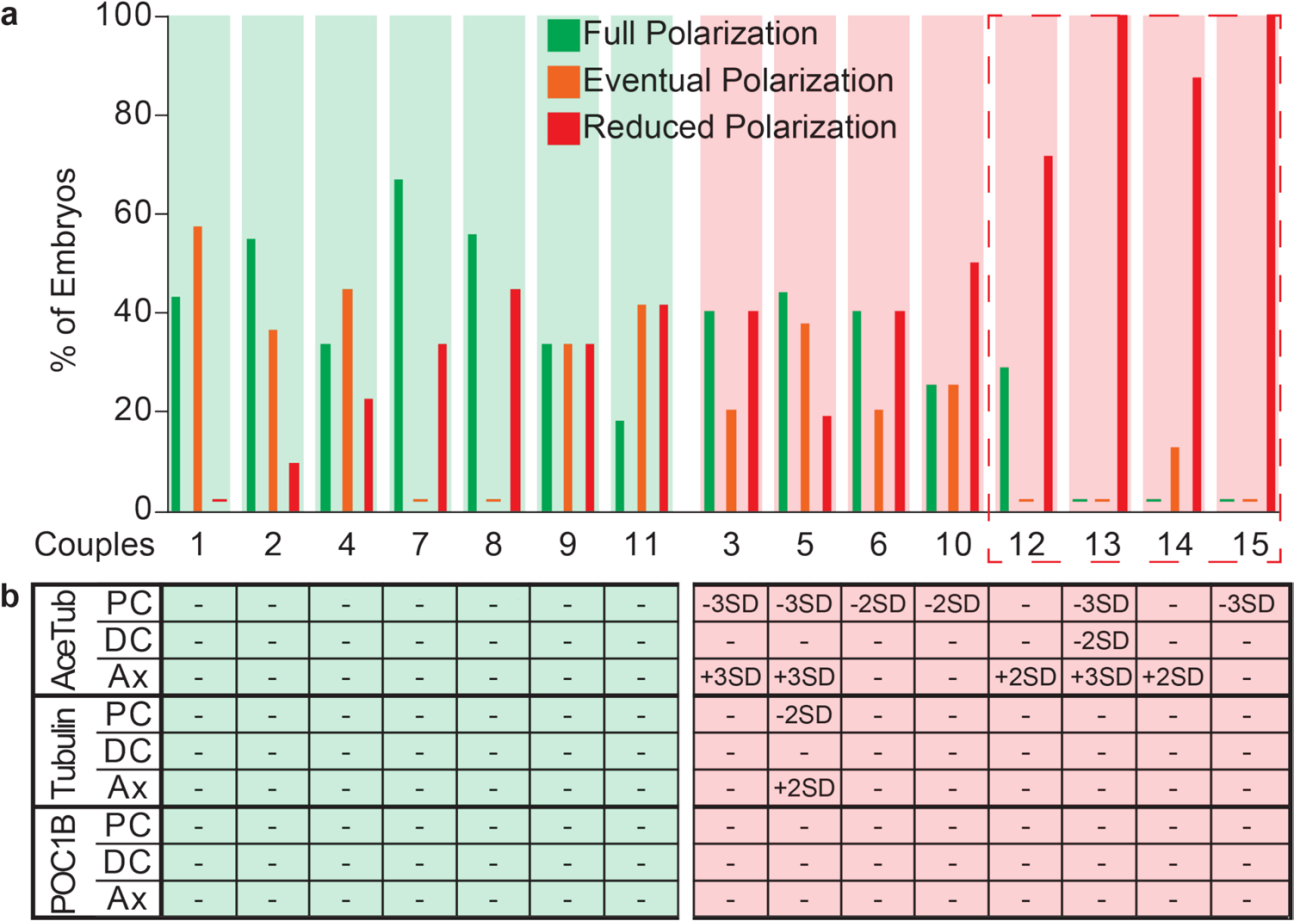
Half of the abnormal FRAC couples had high rates of reduced polarization pattern. **a** Individual breakdown for polarization patterns for each couple. Couples 1, 2, 4, 7, 8, 9, and 11 had normal FRAC scores. Most of their embryos are in either the full (green) or eventual (orange) polarization pattern. Couples 3, 5, 6, 10, 12, 13, 14, and 15 had abnormal FRAC values. Couples 3, 5, 6, and 10 had similar pattern distributions as those in the normal FRAC category. In contrast, couples 12, 13, 14, and 15 had a majority (couples 12 and 14) or all (couples 13 and 15) of their embryos classified as reduced (red) polarization. A dotted red box is placed around the four abnormal FRAC couples with the lowest patterned NPB polarization rates (or the highest rates of the reduced polarization pattern); **b** most outlier values are concentrated in the acetylated tubulin biomarker. PC, proximal centriole; DC, distal centriole; Ax, axoneme

NPB polarization patterns were further examined for each couple to look for predictors of spermatozoa and zygotic defects (Fig. 4a). The couples with normal FRAC values (couples 1, 2, 4, 7, 8, 9, and 11) had similar NPB polarization pattern distributions, with the reduced NPB polarization pattern being at or below 44% (26 ± 17%).

In contrast, couples with abnormal FRAC values had two distinct populations observed. Couples 3, 5, 6, and 10 had a distribution of embryos similar to couples with normal FRAC values, having a reduced NPB polarization embryo pattern at or below 50% (37 ± 13%). Couples 12, 13, 14, and 15 had a distribution of embryos that heavily favored the reduced NPB polarization pattern, with couples 12 and 14 at 71% and 88%, respectively, and couples 13 and 15 at 100% of their embryos ascribed to this pattern. This finding suggests that couples 12, 13, 14, and 15 likely had a spermatozoa centriolar defect that manifested as a zygotic phenotype. Indeed, a *T*-test comparison of the patterned NPB polarization approach of couples 3, 5, 6, and 10 to that of couples 12, 13, 14, and 15 found significant differences in the three patterns and their distribution (Full polarization pattern, *p* = 0.011; eventual polarization pattern, *p* = 0.005; reduced polarization pattern, *p* = 0.001). Importantly, there was not a statistically significant difference between the ages of the two sub-populations in abnormal FRAC couples (couples 3, 5, 6, and 10; 31.5 ± 1.6 years male age and 30.6 ± 3.4 years female age; couples 12, 13, 14, and 15; 33.0 ± 3.0 years male age and 32.2 ± 3.8 years female age; *p* = 0.408, male years, and *p* = 0.555, female years).

No significant differences in the individual NPB polarization time courses were observed between couples 3, 5, 6, and 10 and couples 12, 13, 14, and 15 (Online Resource 6).

We also analyzed the FRAC data to distinguish any biomarker differences between the couples with abnormal FRAC and the subgroups with abnormal or normal NPB polarization (Fig. 4b). Although the majority of outlier values were observed in acetylated tubulin in both subgroups, no biomarker differences that could further separate the two abnormal FRAC subgroups were observed.

### Fifty-six percent of abnormal FRAC embryos were classified as having reduced polarization pattern

**Fig. 5:**
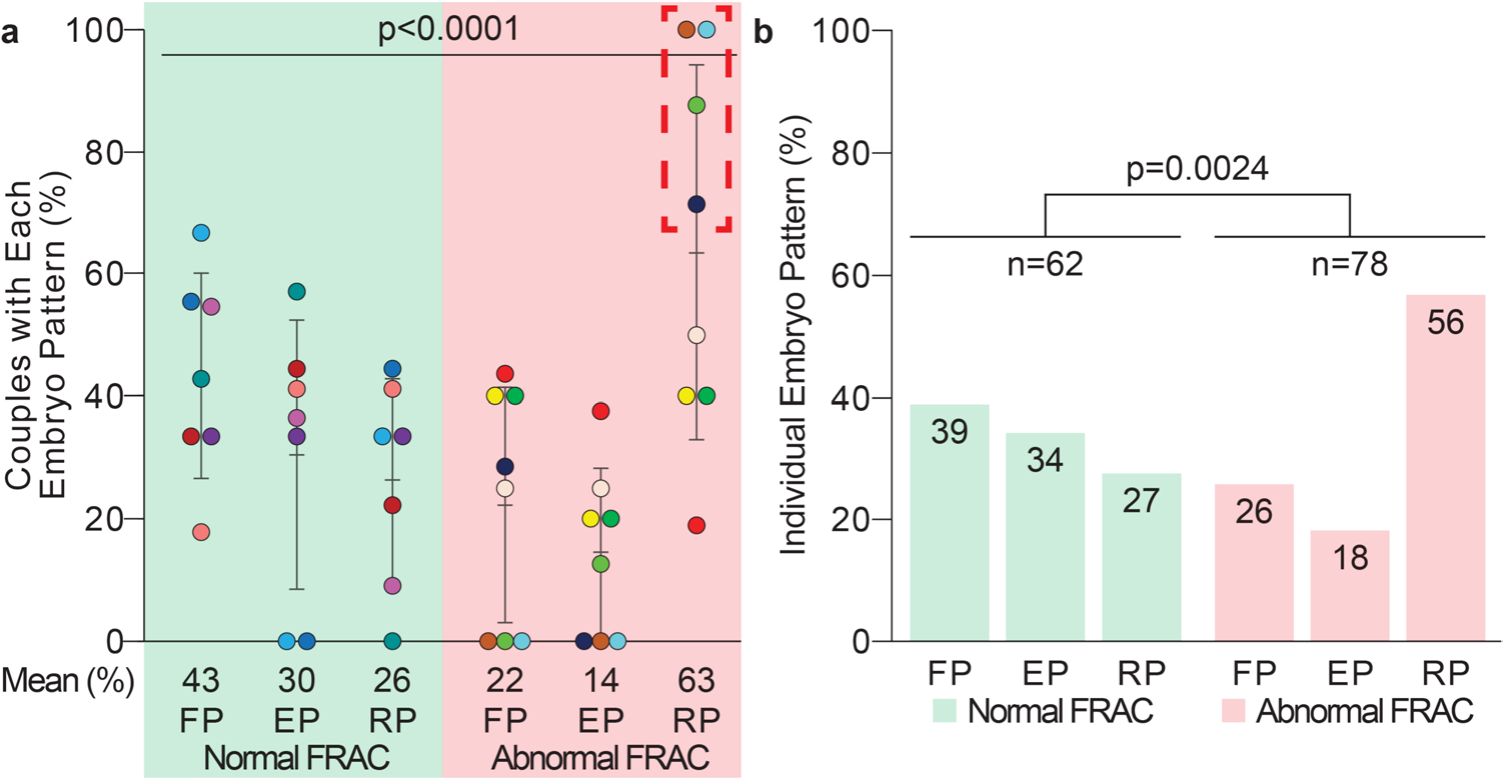
Normal FRAC embryos had fewer reduced polarization pattern rates when compared to abnormal FRAC embryos. **a** Patterned NPB polarization of couples with normal FRAC and abnormal FRAC. Each column represents an average ± 1 SD. Each dot represents a couple (seven per column in normal FRAC and eight per column in abnormal FRAC). Each couple is color-coded to match those in Fig. 2. A dotted red box is placed around the four abnormal FRAC couples with the lowest patterned polarization rate (or the highest rates of the reduced polarization pattern). **b** Patterned NPB polarization of embryos belonging to normal FRAC couples (*n* = 62 embryos) and abnormal FRAC couples (*n* = 78 embryos). Each column represents the overall percentage observed in our study. A chi-square test of independence was used for embryo population analysis. FP, full polarization; EP, eventual polarization; RP, reduced polarization

When examined at a couple level, testing for statistical correlation, patterned NPB polarization was found to be significantly different between couples with normal versus abnormal FRAC values (*p* < 0.0001, chi-square test of independence) (Fig. 5a). A higher rate of reduced polarization patterns in abnormal FRAC couples primarily drives the difference.

The normal FRAC couples had 62 embryos, and abnormal FRAC couples had 78 embryos, for a total of 140 embryos. When examined at an embryo level, patterned NPB polarization was found to be significantly different between the embryos of normal FRAC couples and abnormal FRAC couples (*p* = 0.0024, chi-square test of independence) (Fig. 5b). Analysis of the results found the largest difference in the reduced polarization (RP) pattern between normal (27%, *n* = 17 embryos) and abnormal (56%, *n* = 44 embryos) FRAC couples. Smaller differences were found in the full polarization (FP) and eventual polarization (EP) patterns between normal (FP: 39%, *n* = 24; EP: 34%, *n* = 21) and abnormal (FP: 26%, *n* = 20; EP: 18%, *n* = 14) FRAC couples.

A receiver operating characteristic (ROC) curve test was calculated on the 15 couples for the full, eventual, and reduced polarization scores. The area under the curve (AUC) value for full polarization was 0.786, eventual polarization was 0.732, and reduced polarization was 0.839. An AUC value of 0.7 to 0.8 is moderate, 0.8 to 0.9 is excellent, and > 0.9 is outstanding [43]. Thus, our study found a moderate to excellent correlation.

## Discussion

The couple- and embryo-level analyses of patterned NPB polarization revealed statistically significant differences between patients with normal and abnormal FRAC values. These results suggested that FRAC may identify subtle spermatozoa centriole defects that are functionally significant in the zygote, therefore justifying a large-scale study of the hypothesis that spermatozoa centriole quality determined by FRAC correlates with zygote nucleoli polarization.

A benefit of NPB scoring is that it is a non-invasive and low-cost option for analyzing embryos, explaining its use in previous studies [36–38, 41]. Past studies have used a single, static NPB polarization scoring system, capturing NPB placement in the two pronuclei anywhere from 16 to 18 h post-fertilization (i.e., based on a single image [41, 44]). Recent studies have taken a dynamic approach to track NPB migration speeds but do not record any NPB polarization (i.e., based on multiple images [45, 46]). We, however, performed dynamic NPB polarization scoring based on tracking NPB polarization patterns during zygote development up until the first cleavage. Our method analyzed NPB polarization well outside the 16–18-h timeframe that previous studies used, going as early as 15 h post-fertilization and up to 40 h post-fertilization. This suggests that the patterned NPB polarization scoring method could be helpful in future studies after being explored in a more extensive study as it is more comprehensive than previous scoring methods.

Multiple properties of NPBs can be used to test for the clinical viability of an embryo. Using Scott’s classifications of NPB polarization scoring [44], Arroyo and colleagues [47] showed that highly polarized embryos were more likely to result in good-quality embryos. Faster NPB migration occurred more frequently in euploid zygotes than in aneuploid zygotes, eventually developing into blastocysts [45, 46]. It has also been shown that abnormal NPB morphology (number, size, and distribution) is related to poor-quality embryo implantation and pregnancy via ICSI [36, 41, 48], as well as a low ovarian reserve in the woman [49]. Poor-quality spermatozoa, either from oligospermic or azoospermic men, have led to embryos with degraded NPB polarization in the past [50]. While our exploratory pilot study analyzed NPB distribution/polarization, the size of each NPB was not examined due to the different planes of focus.

Interestingly, our study found that 44% (61/140) of embryos failed to polarize by the time of nuclear envelope breakdown. This is similar to the results of Cavazza and colleagues [37] in that 48% of human embryos and their NPBs failed to cluster by nuclear envelope breakdown. Furthermore, we found four abnormal FRAC couples (3, 5, 6, and 10) with embryo pattern distributions similar to the seven normal FRAC couples. This may mean that while the FRAC assay identifies the spermatozoa centrioles from the males of these four couples as abnormal, patterned NPB polarization scoring suggests that these centrioles function normally in the zygote. The other four abnormal FRAC couples (12, 13, 14, and 15) showed a high percentage of their zygotes in the reduced polarization pattern, suggesting that they have partially dysfunctional centrioles. Couples 12 and 15 had normal semen analysis, while couple 13 had asthenoteratozoospermia, and couple 14 had asthenozoospermia. This difference creates two distinct subgroups within the abnormal FRAC population. This could mean that current FRAC biomarkers are only 50% effective in identifying centriole defects relevant to zygotic centrosome function; these would be couples 12, 13, 14, and 15. To remedy this, we would need to identify a more effective spermatozoa centriole biomarker to stratify further the abnormal FRAC couples between the couples (3, 5, 6, and 10) that look like normal FRAC couples’ embryos and those that mainly have their zygotes belonging to the reduced polarization pattern (couples 12, 13, 14, and 15). Additionally, couple 6, a couple with a male with abnormal centrioles, had one zygote out of ten analyzed with a unique polarization pattern starting with high polarization and ending with low polarization. Couple 6 was identified as having an abnormal FRAC score but had normal NPB polarization patterns. This one embryo could be explained by centrosome drift over time from their positions between the two pronuclei.

When FRAC data is further broken down, most outlier values were found solely in acetylated tubulin, with two outliers in tubulin in the spermatozoa of couple 5. No outlier FRAC values were found in POC1B in the abnormal FRAC couples. This is consistent with our past findings that acetylated tubulin is the biomarker with the most outliers in humans [26] and bovines [25]. This can be explained by the fact that acetylated tubulin is a post-translational modification of tubulin and is sensitive to the overall health of the spermatozoon. Mature spermatozoa have very low transcription and translation, so post-translational modifications may be the main way the spermatozoon can respond to environmental stressors [51, 52].

When patterned NPB polarization scoring and FRAC assay are paired, they can identify subtle differences in NPB polarization between normal and abnormal FRAC couples. This suggests FRAC can identify spermatozoa centriole abnormalities that manifest as embryonic phenotypes. However, a limitation of this pilot study is that it focused solely on the effect of the paternal spermatozoa centrioles on zygotic centrosome function, while the egg may also contribute. Indeed, post-fertilization, centrioles are part of the larger centrosome complex, including egg proteins that help polarize the NPBs in the zygote. This maternal pericentriolar material is recruited by the paternal centrioles, and defective centrioles could have a cascading effect by gathering defective pericentriolar material, which can be seen in cancer cells [53]. In this case, independent or centriole-dependent defective maternal pericentriolar material could cause reduced NPB polarization rates. It is also possible that the pericentriolar material acts as a compensatory mechanism to make up for any centriole defects or even mask defective centrioles. However, no current assays are available to identify abnormalities in the maternal pericentriolar material.

## Conclusions

In conclusion, this pilot study suggests that a male’s spermatozoa centriole quality by FRAC correlates with a couple’s NPB polarization by NPB polarization scoring. This suggests that FRAC can identify centriole anomalies with functional consequences in the zygote. Further FRAC and NPB polarization testing is needed to elucidate the role of these essential structures in zygote development.

## Supplementary Information

Below is the link to the electronic supplementary material.

Supplementary file1 (PDF 31 KB)

Supplementary file2 (PDF 31 KB)

Supplementary file3 (PDF 51 KB)

Supplementary file4 (PDF 299 KB)

Supplementary file5 (PDF 545 KB)

Supplementary file6 (PDF 561 KB)

## Data Availability

The original data presented in the study is openly available at Figshare under the same name as the article.

## Abbreviations

FRAC: Fluorescence-Based Ratiometric Analysis of Sperm Centrioles
NPB: Nucleolus Precursor Bodies

## Declarations

### Patents

The University of Toledo has filed provisional patent application number 63/117,056, with TAR named on this patent based on the original article describing the FRAC method.
