# Supplementary material for: Spermatozoa centriole quality determined by FRAC may correlate with zygote nucleoli polarization—a pilot study
Source: J Assist Reprod Genet. 2025 Feb 7;42(4):1121–32. doi: 10.1007/s10815-025-03411-x (PMC12055725; doi:10.1007/s10815-025-03411-x)
Supplement: Supplementary file 1 — Supplementary file1 (PDF 31 KB) [file 10815_2025_3411_MOESM1_ESM.pdf]

Article Title: Spermatozoa Centriole Quality Determined by FRAC May Correlate with Zygote Nucleoli Polarization – a Pilot Study

Journal Name: *Journal of Assisted Reproduction and Genetics*

Author Names: Derek F Kluczynski, Ankit Jaiswal, Min Xu, Nagalakshmi Nadiminty, Barbara Saltzman, Samantha Schon, Tomer Avidor-Reiss

Corresponding Author: Tomer Avidor-Reiss

Affiliations: Department of Biological Sciences, College of Natural Sciences and Mathematics, University of Toledo, Toledo, OH, USA

Department of Urology, College of Medicine and Life Sciences, University of Toledo, Toledo, OH, USA

Email: tomer.avidorreiss@utoledo.edu

Online Resource 1 The statistical significance of the main hypotheses tested in this study. [1], T-test; **[2]**, Chi-Squared Test of Independence with 2 degrees of freedom

	Couple (n=15)<br>(7 FRAC - & 8 FRAC +)	Embryo (n=140)<br>(62 FRAC - & 78 FRAC +)
Maximal NPB Polarization	P=0.09[1]	P=0.06[1]
Patterned NPB Polarization	P=0.0000008[2]	P=0.0024[2]
