# Supplementary material for: Spermatozoa centriole quality determined by FRAC may correlate with zygote nucleoli polarization—a pilot study
Source: J Assist Reprod Genet. 2025 Feb 7;42(4):1121–32. doi: 10.1007/s10815-025-03411-x (PMC12055725; doi:10.1007/s10815-025-03411-x)
Supplement: Supplementary file 2 — Supplementary file2 (PDF 31 KB) [file 10815_2025_3411_MOESM2_ESM.pdf]

Article Title: Spermatozoa Centriole Quality Determined by FRAC May Correlate with Zygote Nucleoli Polarization – a Pilot Study

Journal Name: *Journal of Assisted Reproduction and Genetics*

Author Names: Derek F Kluczynski, Ankit Jaiswal, Min Xu, Nagalakshmi Nadiminty, Barbara Saltzman, Samantha Schon, Tomer Avidor-Reiss

Corresponding Author: Tomer Avidor-Reiss

Affiliations: Department of Biological Sciences, College of Natural Sciences and Mathematics, University of Toledo, Toledo, OH, USA

Department of Urology, College of Medicine and Life Sciences, University of Toledo, Toledo, OH, USA

Email: tomer.avidorreiss@utoledo.edu

Online Resource 2 Antibodies used in spermatozoa labeling

Target	Name, Company, Catalog Number, Batch Number	Dilution	Role
POC1B	Rabbit anti-POC1B (Thermo Fisher Scientific)	1:100	Primary Antibody
Acetylated Tubulin	Mouse anti-acetylated tubulin (Sigma clone 6-11B-1)	1:100	Primary Antibody
Tubulin	Sheep anti-tubulin (Cytoskeleton, Inc)	1:600	Primary Antibody
Donkey anti-rabbit Alexa 647	Donkey anti-Rabbit IgG (H+L) Highly Cross-Adsorbed Secondary Antibody, Alexa Fluor Plus 647, Thermo Fisher Scientific, A-32795	1:400	Secondary Antibody
Donkey anti-mouse DyLight 488	Mouse IgG (H+L) Cross-Adsorbed Secondary Antibody, Thermo Fisher Scientific, SA5–10166	1:400	Secondary Antibody
Donkey anti-sheep Alexa 555	Donkey anti-Sheep Alexa 555, Thermo Fisher Scientific	1:100	Secondary Antibody
