# Supplementary material for: Spermatozoa centriole quality determined by FRAC may correlate with zygote nucleoli polarization—a pilot study
Source: J Assist Reprod Genet. 2025 Feb 7;42(4):1121–32. doi: 10.1007/s10815-025-03411-x (PMC12055725; doi:10.1007/s10815-025-03411-x)
Supplement: Supplementary file 3 — Supplementary file3 (PDF 51 KB) [file 10815_2025_3411_MOESM3_ESM.pdf]

Article Title: Spermatozoa Centriole Quality Determined by FRAC May Correlate with Zygote Nucleoli Polarization – a Pilot Study

Journal Name: *Journal of Assisted Reproduction and Genetics*

Author Names: Derek F Kluczynski, Ankit Jaiswal, Min Xu, Nagalakshmi Nadiminty, Barbara Saltzman, Samantha Schon, Tomer Avidor-Reiss

Corresponding Author: Tomer Avidor-Reiss

Affiliations: Department of Biological Sciences, College of Natural Sciences and Mathematics, University of Toledo, Toledo, OH, USA

Department of Urology, College of Medicine and Life Sciences, University of Toledo, Toledo, OH, USA

Email: tomer.avidorreiss@utoledo.edu

Online Resource 3 Couples' semen analysis and infertility diagnosis

Couple Number	Male Age (years)	Female Age (years)	Semen Analysis	Infertility Diagnosis
**Couples with Normal FRAC Men**				
1	34.6	31.0	Normal	Embryo banking - fertility preservation
2	26.7	27.1	Normal	Endometriosis, Ovulatory Dysfunction
4	36.8	36.8	Asthenozoospermia	Male Factor, Ovulatory Dysfunction, Bilateral Varicoceles
7	39.1	35.5	Astheno-teratozoospermia	Male Factor, Diminished Ovarian Reserve
8	31.9	36.3	Teratozoospermia	Male Factor, Ovulatory Dysfunction
9	28.9	29.1	Oligo-teratozoospermia	Male Factor, Ovulatory Dysfunction
11	38.7	36.2	Normal	Diminished Ovarian Reserve, Polycystic Ovary Syndrome
**Couples with Abnormal FRAC Men**				
3	31.3	32.1	Oligoasthenoteratozoospermia	Male Factor, Polycystic Ovary Syndrome
5	32.9	31.9	Asthenoteratozoospermia	Male Factor, Ovulatory Dysfunction
6	29.3	25.5	Oligoasthenozoospermia	Male Factor, Polycystic Ovary Syndrome
10	32.4	32.6	Normal	Recurrent Loss Pregnancy, Balanced Translocation
12	30.1	27.3	Normal	Polycystic Ovary Syndrome
13	30.8	33.3	Asthenoteratozoospermia	Male Factor
14	34.9	31.5	Asthenozoospermia	Endometriosis, Diminished Ovarian Reserve, Recurrent Loss Pregnancy
15	36.1	36.5	Normal	Diminished Ovarian Reserve, Tubal Disease
