# Supplementary material for: Spermatozoa centriole quality determined by FRAC may correlate with zygote nucleoli polarization—a pilot study
Source: J Assist Reprod Genet. 2025 Feb 7;42(4):1121–32. doi: 10.1007/s10815-025-03411-x (PMC12055725; doi:10.1007/s10815-025-03411-x)
Supplement: Supplementary file 4 — Supplementary file4 (PDF 299 KB) [file 10815_2025_3411_MOESM4_ESM.pdf]

Article Title: Spermatozoa Centriole Quality Determined by FRAC May Correlate with Zygote Nucleoli Polarization – a Pilot Study

Journal Name: *Journal of Assisted Reproduction and Genetics*

Author Names: Derek F Kluczynski, Ankit Jaiswal, Min Xu, Nagalakshmi Nadiminty, Barbara Saltzman, Samantha Schon, Tomer Avidor-Reiss

Corresponding Author: Tomer Avidor-Reiss

Affiliations: Department of Biological Sciences, College of Natural Sciences and Mathematics, University of Toledo, Toledo, OH, USA

Department of Urology, College of Medicine and Life Sciences, University of Toledo, Toledo, OH, USA

Email: tomer.avidorreiss@utoledo.edu

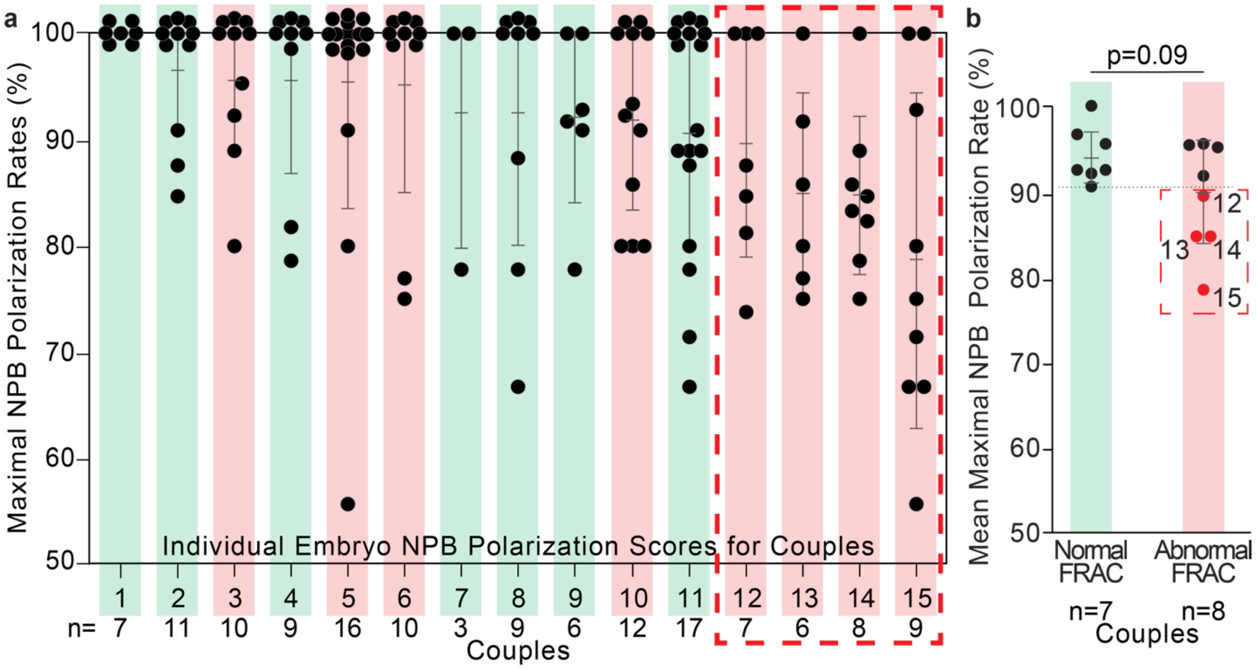

Online Resource 4 Pre-analysis of couples with abnormal FRAC values have lower maximal NPB polarization that is not statistically different. (**a**) Individual maximal NPB polarization scores for couples with normal FRAC (green bars) and abnormal FRAC (red bars). Black dots represent individual maximal NPB polarization scores from embryos. A dotted red box surrounds the four abnormal FRAC couples with the lowest maximal NPB polarization rates. (**b**) The mean maximal NPB Polarization rates of Couples with Normal FRAC men and Couples with Abnormal FRAC men had a p-value of 0.090. Couples 1, 2, 4, 7, 8, 9, and 11 are shown in the Normal FRAC column, with all their scores falling above 91%. Couples 3, 5, 6, 10, 12, 13, 14, and 15 are shown in the Abnormal FRAC column, with half of the couples (3, 5, 6, and 10) falling above this 91% cutoff while the other half of couples (12, 13, 14, and 15, red dotted red box) fell below this cutoff and are denoted as red X's. For maximal Nucleolus Precursor Body polarization scoring, we analyzed and captured the highest NPB polarization score (see the equation in the Results section), regardless of the development time
