# Supplementary material for: Spermatozoa centriole quality determined by FRAC may correlate with zygote nucleoli polarization—a pilot study
Source: J Assist Reprod Genet. 2025 Feb 7;42(4):1121–32. doi: 10.1007/s10815-025-03411-x (PMC12055725; doi:10.1007/s10815-025-03411-x)
Supplement: Supplementary file 5 — Supplementary file5 (PDF 545 KB) [file 10815_2025_3411_MOESM5_ESM.pdf]

Article Title: Spermatozoa Centriole Quality Determined by FRAC May Correlate with Zygote Nucleoli Polarization – a Pilot Study

Journal Name: *Journal of Assisted Reproduction and Genetics*

Author Names: Derek F Kluczynski, Ankit Jaiswal, Min Xu, Nagalakshmi Nadiminty, Barbara Saltzman, Samantha Schon, Tomer Avidor-Reiss

Corresponding Author: Tomer Avidor-Reiss

Affiliations: Department of Biological Sciences, College of Natural Sciences and Mathematics, University of Toledo, Toledo, OH, USA

Department of Urology, College of Medicine and Life Sciences, University of Toledo, Toledo, OH, USA

Email: tomer.avidorreiss@utoledo.edu

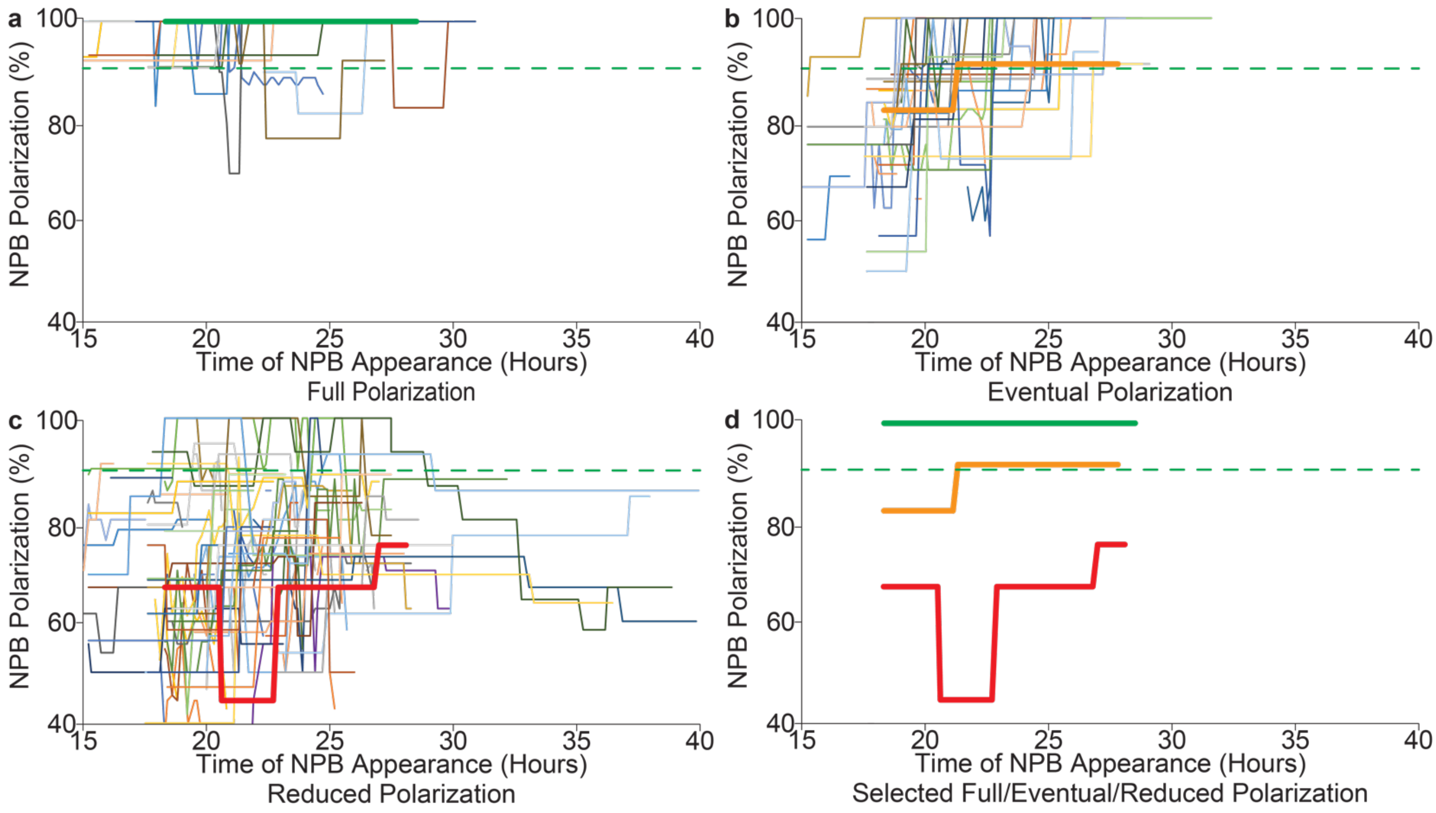

Online Resource 5 Three NPB polarization patterns were observed. (**a**) A full polarization pattern was characterized by scoring starting above the 91% cutoff and ending above the cutoff. Some deviation occurs, which makes the scoring fall below the cutoff at times during the patterned NPB polarization scoring. N=44 embryos analyzed. The thick green line represented an example of the full polarization pattern; (**b**) An eventual polarization pattern was characterized by scoring starting below the 91% cutoff but ending above the cutoff at the time of NPB disappearance. N=35 embryos analyzed. The thick orange line represented an example of the eventual polarization pattern; (**d**) A reduced polarization pattern was characterized by scoring starting and ending below the 91% cutoff. N=61 embryos analyzed. The graphs do not show three embryos with intervals below 40% polarized. The thick red line represented an example of the reduced polarization pattern; (**d**) A graph of a selected embryo from each pattern with similar start and end times
